# Crystal structure of HLA-B*5801, a protective *HLA allele* for HIV-1 infection

**DOI:** 10.1007/s13238-016-0309-y

**Published:** 2016-09-09

**Authors:** Xiaolong Li, Pedro A. Lamothe, Robert Ng, Shutong Xu, Maikun Teng, Bruce D. Walker, Jia-huai Wang

**Affiliations:** 1 School of Life Sciences, University of Science and Technology of China, Hefei, 230027 China; 2 Department of Medical Oncology and Cancer Biology, Dana-Farber Cancer Institute, Harvard Medical School, Boston, MA 02215 USA; 3 Ragon Institute of Massachusetts General Hospital, Massachusetts Institute of Technology, Harvard University, Boston, MA 02139 USA; 4 Howard Hughes Medical Institute, Chevy Chase, MD 20815 USA; 5 Department of Pediatrics and Department of Biological Chemistry and Molecular Pharmacology, Harvard Medical School, Boston, MA 02115 USA; 6 Biomarin Pharmaceutical, 790 Lincoln Ave, San Rafael, CA 94901 USA

**Dear Editor,**

Conquering the AIDS epidemic remains one of the most challenging global health issues. Despite impressive advances in combination antiretroviral therapy (cART) to treat HIV, and substantial progress in the development of prevention interventions, a protective vaccine has remained elusive. Successful vaccination will require the immune system to function better than it does in natural infection, in which the infection invariably becomes chronic. However, there are a very small number of patients termed “elite controllers” who become infected but can control viremia, with the preponderance of evidence pointing to CD8^+^ T cell mediated immune control. The existence of such patients implies that the human immune system, although not able to eradicate infection, is potentially capable of durable control of HIV-1 and prevention of HIV-associated disease [reviewed in (Deeks and Walker, 2007; Walker and Yu, 2013)].

The beauty of Major Histocompatibility Complex (MHC)-restriction in T cell immunity is that within a population, there may always be individuals who bear particular *MHC alleles*, able to present antigenic peptides derived from invading pathogens for T cell receptors (TCRs) to recognize. This ultimately gives rise to effective cellular immunity to control chronic infection to save the species (Zinkernagel and Doherty, 1997). This has been exemplified by the global HIV-1 epidemic. The above-mentioned “elite controllers” (as opposed to progressors) are enriched in expression of certain “protective” class I Human Leukocyte Antigen (HLA) alleles such as HLA-B*57/5801 subtypes and B*27. By contrast, other persons who express so-called deleterious or disease susceptible alleles, including HLA-B*18/*53* are much more likely to develop AIDS in the absence of cART (O’Brien et al., 2001). Obviously, the differential peptide presentation by HLA molecules is rooted in host MHC polymorphism. Indeed, a genome-wide association study (GWAS) clearly demonstrated that specific amino acid variations in the peptide-binding groove are associated with host control of HIV-1 infection (Pereyra et al., 2010). Therefore, unraveling the molecular mechanism of host control of HIV-1 infection by cellular immunity is extremely important for eventually devising a strategy to eliminate the HIV-1 epidemic.

To begin to address the structural basis for T cell mediated control of HIV, we initiated a structural analysis of an immunodominant CD8^+^ T cell epitope QW9 (QASQEVKNW, p24^gag^ residues 176–184) from HIV-1 bound to the restricting HLA class I molecule B*5801. This epitope is of particular interest because the conserved Gag protein forms the essential capsid of HIV-1 (Zhao et al., 2013), with mutations likely to impose a fitness cost. In addition, QW9 can be presented by a spectrum of HLA molecules for comparative investigation (Buseyne et al., 2001; Buseyne et al., 1997; Currier et al., 2005; Kloverpris et al., 2013). Here, we report the first crystal structures of HLA-B*5801, one of the most HIV-protective HLA molecules, loaded with QW9 and its natural variants QW9-S3T and QW9-E5D. All three structures are in a very similar crystal lattice and have two independent molecules in one asymmetric unit. Fig. 1A is the overall view of one of the QW9wt/HLA-B*5801 complexes in crystal structure. The most interesting feature of these three QW9/HLA-B*5801 complex structures is that the conformation of the middle portion of the bound peptide appears varied (Fig. 1B), even between the two independent molecules in the same crystal structure (Fig. 1C–E). It is not uncommon to have a highly exposed residue at the center of the presenting peptide on the HLA molecule to have its side chain flexible (Ladell et al., 2013). Our unique observation is that among these six QW9/HLA complexes in three crystal structures, the peptide residue K7 can be modeled outwards in two cases (Fig. 1C for QW9wt-1 and Fig. 1D for QW9-E5D-2), but buried inwards in another two cases (Fig. 1C for QW9wt-2 and Fig. 1E for QW9-S3T-2). It should be emphasized that despite the weak density of the long and floppy side chain, the density for the main chain conformation and the Cα-Cβ direction of the K7 residue in Fig. 1C–E is actually convincing enough for the assignment of its side chain to be outwards (convex-shaped main chain in Fig. 1C for QW9wt-1 and Fig. 1D for QW9-E5D-2) or inwards (concave-shaped main chain in Fig. 1C for QW9wt-2 and Fig. 1E for QW9-E5D-2).

**Figure 1:**
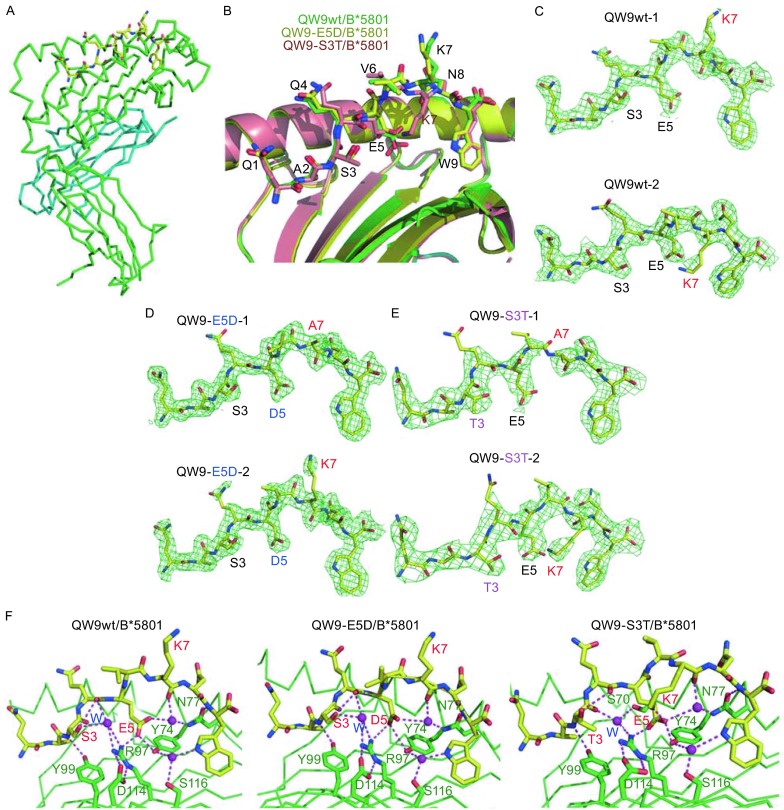
Structures of HLA-B*5801 complex with QW9 and its variants. (A) Overall view of the structure of QW9wt/HLA-B*5801. The heavy chain is in green and the β2m domain is in cyan. The peptide QW9wt is in a yellow stick model. (B) Overlay of structures of HLA-B*5801 loaded with wild-type QW9 and its variants, E5D and S3T. The front helix of HLA has been removed for clarity. (C–E) The 2Fo-Fc difference maps for the bound peptide only. Shown here are peptides in six independent peptide/HLA-B*5801 complexes of three crystal structures determined. The map is contoured at 0.9 σ level. Note that in Fig. D and E, the densities of QW9-E5D-1 and QW9-S3T-1 are too weak to assign the long floppy side chain of K7. Only Ala is modeled instead. But their main chain conformations are in convex shape, indicating that the flexible side chain of K7 should point outwards. (F) The peptide-binding mode. Shown here is the hydrogen-bonding network between peptide and HLA molecule for three representative structures. Hydrogen bonds are shown in magenta dash lines. The magenta-colored balls are water molecules. HLA-B*5801 and its residues are in green and QW9 is in yellow stick model. The important residues on QW9 and the variants are labeled in red. Again the front helix of HLA has been removed for clarity

The above structural data imply that unlike those anchoring residues (W9 in QW9 as a typical example, see Fig. 1B), the conformation of the central protruding residues, K7 in particular, can actually be in a dynamic distribution. This is probably because the whole protruding and mobile portion of the peptide (residues 6 to 8) gives the central K7 enough room for flexibility. It is possible that during the peptide loading process, the K7 residue can populate in statistically different conformations, either fluttering on the peptide/HLA surface or embedded in the anchoring pocket of the peptide-binding groove. Fig. 1C–E reflect what are captured in crystallization. It should be extremely interesting to explore, in the future, how the dynamic features of this peptide presentation by its restricting HLA molecule would affect TCR recognition in the context of host control of HIV-1 infection.

The side chain of QW9 residue E5 is fully buried inside the binding-groove, whereas residue S3 has its hydroxyl group semi-exposed (Fig. 1B and 1F). Fig. 1F demonstrates the QW9 specific binding mode in the peptide-binding groove of HLA-B*5801 in three representative complexes. Illustrated in this figure is a hydrogen-bonding network, mediated by three conserved bound water molecules within the groove. Comparing E5 in the structure of QW9wt/HLA-B*5801 (Fig. 1F, left panel) with D5 in the structure of QW9-E5D/HLA-B*5801 (Fig. 1F, middle panel), they both form three hydrogen bonds in the binding groove. Apparently the mutation of E5D does not change this binding pattern. The mutation S3T is somewhat complicated. First of all, the replacement of S3 to T3 does not noticeably change the location the hydroxyl group, as shown in Fig. 1B. The “extra” methyl group in QW9-S3T/HLA-B*5801 appears buried and occupies a space near the bound water labeled as a blue “W” in Fig. 1F (right panel). Comparing QW9wt/HLA-B*5801 (Fig. 1F, left panel) with QW9-S3T/HLA-B*5801 (Fig. 1F, right panel), this “extra” methyl group does not seem to affect the overall conformation of QW9/HLA-B*5801. In other words, from a structural point of view, this “extra” methyl group in S3T mutation does not seem to affect peptide presentation. To sum up, we may have provided a structural rationale as to why the naturally occurring E5D and S3T mutations would not be expected to significantly alter presentation and recognition of epitope QW9 presented by HLA-B*5801 (Navis et al., 2007).

The genes coding for the heavy chain of HLA molecules are highly polymorphic, which determines differential peptide-presentation and TCR-recognition. The sequence variation between the HIV-1 “protective” allele HLA-B*5801 and the deleterious allele HLA-B*5301 is eight residues (Fig. 2A), which cluster on the α1-helical region of the heavy chain shown in red in Fig. 2B. Fig. 2C and 2D demonstrate the electrostatic potential surface representation for HLA-B*5801 and HLA-B*5301, respectively. The view is down from the TCR perspective, identical as the ribbon drawing of HLA-B*5801 shown on Fig. 2B. As expected, the most remarkable distinction between HLA-B*5801 and HLA-B*5301 is at the top left region (shown as a circle in Fig. 2C and 2D), corresponding to the sequence variable area between HLA-B*5801 and HLA-B*5301 (the middle portion difference between Fig. 2C and 2D was basically from the bound peptides). This is the region that is contacted more by the TCR complementary-determining region 1α (CDR1α loop and also the CDR3α loop in a common pMHC/TCR docking mode that was first identified in 1998 (Teng et al., 1998). This canonical binding mode is now seen in over 50 unique pMHC/TCR structures determined. The TCR will use its germline-derived CDR1 and CDR2 loops to contact genetically encoded polymorphic helical regions of MHC molecule, and its V(D)J-recombined CDR3 loop in the center of the complex for antigenic peptide recognition (Adams et al., 2016).

**Figure 2:**
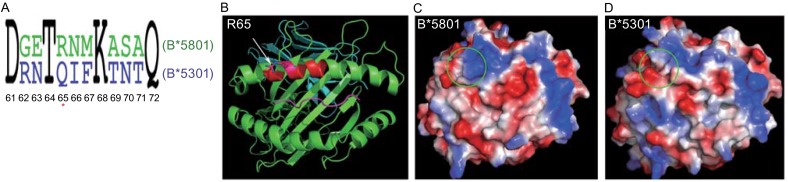
Comparison between HLA-B*5801 and HLA-B*5301. (A) Sequence alignment of HLA-B*5801 and HLA-B*5301 (residues 61–72). Only eight residues differ between the two HLA molecules, and the residue R65 which contributes to the large positive area in HLA-B*5801 is labeled with a star. (B) Ribbon drawing of HAL-B*5801 molecule viewing down the peptide-binding groove from the TCR perspective. The distinct residues between HLA-B*5801 and HLA-B*5301 are in red region and cluster at the α1-helical region of HLA molecule. The R65 is labeled and highlighted as sticker here. (C and D) Electrostatic potential surface representation in the same view as in the ribbon drawing in B, for HLA-B*5801 and HLA-B*5301 (PDB: 1A1M), respectively. The red color represents negative potential, the blue positive and the while color neutral. The most remarkable distinction between HLA-B*5801 and HLA-B*5301 is highlighted as a circle

In T cell immunity, MHC-restriction means that a TCR not only recognizes antigenic peptide, but also directly interacts with the specific MHC molecule that presents this particular peptide. The surface representation shown in Fig. 2C and/or 2D is the combined recognition surface, and the top left corner difference between these two figures is what HLA-B*5801 and HLA-B*5301 would differentially contribute to stimulate disparate TCR molecules. It should be emphasized that the exposed R65 in HLA-B*5801 (star-marked in Fig. 2A and shown in Fig. 2B as a stick model) as opposed to Q65 in HLA-B*5301 must be the important factor to give rise to the large positive area in the region for HLA-B*5801. This area is more likely to be contacted by CDR1α loop of a TCR, a germline-derived structural element. Our immunological data show that even sequences encoding the CDR3 loop were significantly more “germline-like’ used by controllers than those used by progressors (Chen et al., 2012). The advantage here may be related to a greater ability to recognize mutational variants of an epitope. These should all contribute to the host control of HIV-1 infection. It should also be emphasized that more of the 8-residue differences between HLA-B*5801 and HLA-B*5301 are involved in peptide-binding, which may not show up on the surface and hence not be directly engaged in TCR contacts. These HLA residues determine which peptide to present. While some distinct HLA-alleles can present the identical peptide, the polymorphism may cause disparate HLA molecules to have different peptide binding affinities and binding kinetics, which will also affect TCR recognition (Kloverpris et al., 2013).

In summary, we have reported the first crystal structure of HLA-B*5801 loaded with an immunodominant antigenic peptide QW9 derived from HIV Gag protein. The unique observation is that the central peptide residue K7 can assume either a buried conformation in peptide-binding groove or an exposed one. This dynamic may affect TCR recognition. We have also provided a structural rationale for why natural mutations of E5D and S3T can elicit a similar T cell response as the wild type QW9 does. Comparing the surface representation between HLA-B*5801 and HLA-B*5301, we have discussed how these two HLA alleles may contribute differently to host control of HIV-1 infection.

## Electronic supplementary material

Below is the link to the electronic supplementary material.

Supplementary material 1 (PDF 212 kb)
