# Supplementary material for: Crystal structure of HLA-B*5801, a protective HLA allele for HIV-1 infection
Source: Protein Cell. 2016 Sep 9;7(10):761–5. doi: 10.1007/s13238-016-0309-y (PMC5055491; doi:10.1007/s13238-016-0309-y)
Supplement: Supplementary file 1 — Supplementary material 1 (PDF 212 kb) [file 13238_2016_309_MOESM1_ESM.pdf]

SUPPLEMENTARY MATERIALS

Structures of QW9/HLA-B*5801 and the flexibility of the QW9 central residue K7

We determined crystal structures of HLA-B*5801 loaded with wild-type QW9, QW9wt, as well as variants QW9-S3T and QW9-E5D, which are known to arise *in vivo*. Table S1 lists the crystallographic statistics of these structures. From the overall view of the QW9wt/HLA-B*5801 complex, the peptide-binding site of a typical class I HLA molecule contains 6 binding pockets (termed A through F) in a groove-like region of the HLA molecule. In the case of QW9, the C-terminal residue of this peptide is tryptophan (W9), which has a bulky hydrophobic side chain containing an indole ring buried in the hydrophobic F pocket of the B*5801 HLA molecule. The amino terminus of the peptide anchors into the peptide-binding pocket A with a number of hydrogen bonds to the amide group (Saper et al., 1991). The distance between the two Cα atoms of the two terminal residues is then roughly fixed. Any antigenic peptide longer than 8 residues will have its middle portion (often the residues 5-8 near the C-terminus) protruding out of the HLA peptide-binding groove (Stern and Wiley, 1994). This is usually the focal point for TCR contact (Rudolph et al., 2006; Wang and Reinherz, 2012).

From the superposition of three representative structures with the bound peptides in a stick model (Fig. 1B, in main text), it is obvious that only peptide residues from V6 to N8 appear to assume different conformations. At the tip of this segment is the residue K7, which is particularly prominent. The most striking observation is that in one complex like QW9-S3T/HLA-B*5801 shown here this K7 has this side chain buried in the peptide-binding groove of HLA molecule, whereas in the other two complexes such as QW9-wt and QW9-E5D in this figure the K7 is exposed toward the outside. Indeed, for the six HLA molecules in the three crystal structures, this residue K7 has relatively weak electron density for its side chain, indicating its flexibility. The

2Fo-Fc difference maps (Fig. 1C-E, in main text) are contoured at 0.9 σ level in order to define the K7 side chain. From the crystallographic point of view, it is weak because usually the contour level would be set to 1.2-1.5 σ. Even at this 0.9 σ level, in one of the QW9-E5D/HLA-B*5801 (Fig. 1D, in main text) and QW9-S3T/HLA-B*5801 (Fig. 1E, in main text) complexes each, there is no density for definite assignment of the conformation of this K7 side chain. Hence we only model it as Ala, A7.

For class I HLA molecules, to select stably bound peptide for presentation on the cell surface for many hours or even days is an evolutionary advantage (Elliott and Williams, 2005), and *in vitro* reconstitution of the HLA molecule can be efficiently reassembled only in the presence of MHC-restricted peptide (Silver et al., 1991). This suggests that the cognate peptide-loading is part of the HLA refolding process. Our structural data imply that unlike those anchoring residues (W9 in QW9 as a typical example, see Fig. 1C-E, in main text), the conformation of the central protruding residues, K7 in particular, can actually be in a dynamic distribution.

MATERIALS AND METHODS

Cloning

The full DNA sequence of the HLA-B*5801 heavy chain is from EMBL-EBI IPD-IMGT/HLA, and amino acid codons of the HLA-B*5801 ecto-domains were optimized for the bacterial *E.coli* BL21(DE3) expression system. The synthesized gene with a stop codon was inserted into the cloning site of the pUC57 vector, between NdeI and XhoI. Then the HLA-B*5801/pUC57 plasmid was transferred into *E.coli* Top10 competent cells for amplification and then extracted by using Qiagen kit. Instead of PCR, the HLA-B*5801 gene fragment was cut off from the HLA-B*5801/pUC57 plasmid by Ndel and XhoI restriction enzymes and separated by agarose gel electrophoresis. Eventually, the HLA-B*5801 DNA fragment was integrated into the expression vector pET22b(+) at the same cloning site.

Expression and purification of inclusion bodies

Recombinant HLA-B*5801 inclusion bodies were produced in *E.coli* BL21(DE3) cells harboring the HLA-B*5801/pET22b(+) expression plasmid. When the *E.coli* cell density OD_{600} reached 0.6-0.8, inclusion body expression were induced with a final concentration of 1 mM IPTG for 4 hours at 37 ℃ (Stewart-Jones et al., 2003). Cells pellets were harvested and then suspended in the extraction buffer (50 mM Tris-HCl, 100 mM NaCl, 10 mM $MgCl_2$, 1% Triton-X100, pH 8.2) mixed with fresh lysoenzyme, DNase-I and PMSF. After sonification, inclusion bodies were collected at 8000 rpm. To sufficiently lyse cells and make inclusion bodies purer, we resuspended the inclusion bodies and repeated the lysis step one more time. Then the inclusion bodies were washed 3 times with wash buffer (50 mM Tris, 20 mM EDTA, pH 8.0) to further remove Mg^{2+},

detergent and soluble proteins including the enzymes added. The light chain, β2m construct, was from the Barbara Uchańska-Zieger's lab (Institut für Immungenetik, Charité-Universitätsmedizin Berlin, Freie Universität Berlin, Berlin, Germany). Expression and purification methods for β2m were the same as for heavy chain. The purified inclusion bodies were dissolved in 8 M urea and stored at -20 ℃.

Refolding and purification of HLA

Specific peptide is essential for HLA refolding. The peptide QW9 and its variants in this study were synthesized by United BioSystems Inc, and were dissolved in DMSO before refolding. To refold those proteins, HLA-B*5801 (56 mg), β2m (28 mg) and peptide (10 mg) were diluted in 100 mM Tris-HCl pH 8.0, 0.4 M arginine, 0.5 mM oxidized glutathione, 1.5 mM reduced glutathione, 2 mM EDTA, 4 M urea, 0.2 mM PMSF in a volume of 500 ml over 24 hours at 4 ℃. The refolding solution was then dialysed for 4 hours against 0.1 M urea, 10 mM Tris-HCl pH 8.0, and overnight against 10 mM Tris-HCl pH 8.0 at 4 ℃ using a 6-8 kDa molecular mass cut-off dialysis membrane (Spectrum) (Illing et al., 2012). After dialysis, concentrated sample was loaded onto a superdex75 (GE Health) gel filtration column for separation and the correctly refolded HLA-B*5801 protein fraction appeared from 57 ml in 10 mM Tris-HCl pH 8.0, 100 mM NaCl. In addition, the Mono-Q ion exchange column was used in the final step purification.

Crystallization

HLA-B*5801 proteins loaded with QW9 or its variants were concentrated to 7-10 mg/ml in 10 mM Tris-HCl buffer, pH 8.0. Hampton kits were selected as initial screen conditions, and crystals were obtained at room temperature by using the sitting drop vapor diffusion method from 0.1 M sodium citrate tribasic dihydrate pH 5.6, 20% v/v 2-propanol, 20% w/v polyethylene glycol

(PEG) 4,000. We optimized the conditions and found that crystals from 15-20% w/v PEG 4000, 20% w/v 2-propanol, 0.1 M MES pH 6.5 are the best. A drop contained 0.1 μl protein solution mixed with 0.1μl reservoir solution could form crystals within 3 days. The robots NT8 and Rack Imager made by Formulatrix were employed in both crystallization condition screening and optimization.

Data collection, processing and refinement of crystal structures

Diffraction data were collected from cryo-cooled crystals to a resolution range from 2.1 to 2.9 Å (see Table S1) at the APS, Argonne National Laboratories, 19ID beam-line and the ADSC Quantum 315 X-ray diffraction detector. The cryo-protectant solution we used was the crystallization buffer plus 10-20% PEG 400. Diffraction data were processed with the program HKL2000 (Otwinowski and Minor, 1997) and CCP4i (Winn et al., 2011), and molecular replacement was carried out using Phaser in the PHENIX Program Suite (Adams et al., 2010). The search model for all of the three structures is 1A1M from the Protein Data Bank. Structure refinement was also performed in PHENIX with XYZ coordinates, real-space, rigid body, individual B-factor, occupancies and CNS refinement. And the resulting models were manually inspected and modified with the program COOT (Emsley et al., 2010).

REFERENCE

Adams, P.D., Afonine, P.V., Bunkoczi, G., Chen, V.B., Davis, I.W., Echols, N., Headd, J.J., Hung, L.W., Kapral, G.J., Grosse-Kunstleve, R.W., *et al.* (2010). PHENIX: a comprehensive Python-based system for macromolecular structure solution. Acta Crystallogr D Biol Crystallogr *66*, 213-221.

Elliott, T., and Williams, A. (2005). The optimization of peptide cargo bound to MHC class I molecules by the peptide-loading complex. Immunol Rev *207*, 89-99.

Emsley, P., Lohkamp, B., Scott, W.G., and Cowtan, K. (2010). Features and development of Coot. Acta Crystallogr D Biol Crystallogr *66*, 486-501.

Illing, P.T., Vivian, J.P., Dudek, N.L., Kostenko, L., Chen, Z., Bharadwaj, M., Miles, J.J., Kjer-Nielsen, L., Gras, S., Williamson, N.A., *et al.* (2012). Immune self-reactivity triggered by drug-modified HLA-peptide repertoire. Nature *486*, 554-558.

Otwinowski, Z., and Minor, W. (1997). Processing of X-ray diffraction data collected in oscillation mode. In Macromolecular Crystallography, C.W. Carte Jr., and R.M. Sweet, eds. (San Diego, London, Boston, New York, Sydney, Tokyo, Toronto: Academic Press), pp. 307-326.

Rudolph, M.G., Stanfield, R.L., and Wilson, I.A. (2006). How TCRs bind MHCs, peptides, and coreceptors. Annu Rev Immunol *24*, 419-466.

Saper, M.A., Bjorkman, P.J., and Wiley, D.C. (1991). Refined structure of the human histocompatibility antigen HLA-A2 at 2.6 A resolution. J Mol Biol *219*, 277-319.

Silver, M.L., Parker, K.C., and Wiley, D.C. (1991). Reconstitution by MHC-restricted peptides of HLA-A2 heavy chain with beta 2-microglobulin, in vitro. Nature *350*, 619-622.

Stern, L.J., and Wiley, D.C. (1994). Antigenic peptide binding by class I and class II histocompatibility proteins. Structure *2*, 245-251.

Stewart-Jones, G.B., McMichael, A.J., Bell, J.I., Stuart, D.I., and Jones, E.Y. (2003). A structural basis for immunodominant human T cell receptor recognition. Nat Immunol *4*, 657-663.

Wang, J.H., and Reinherz, E.L. (2012). The structural basis of alphabeta T-lineage immune recognition: TCR docking topologies, mechanotransduction, and co-receptor function. Immunol Rev *250*, 102-119.

Winn, M.D., Ballard, C.C., Cowtan, K.D., Dodson, E.J., Emsley, P., Evans, P.R., Keegan, R.M., Krissinel, E.B., Leslie, A.G., McCoy, A., *et al.* (2011). Overview of the CCP4 suite and current developments. Acta Crystallogr D Biol Crystallogr *67*, 235-242.

Table S1 Data collection and refinement statistics (molecular replacement)

HLA-B*5801<br>PDB ID	QW9wt<br>5IM7	QW9-E5D<br>5IND	QW9-S3T<br>5INC
**Data collection**			
Wavelength (Å)	0.9793	0.9793	0.9793
Resolution range (Å)	50.00 - 2.50<br>(2.59 - 2.50)	50.00 - 2.11<br>(2.15 - 2.11)	50.00 - 2.90<br>(2.95 - 2.90)
Space group	$P\ 2_1\ 2_1\ 2_1$	$P\ 2_1\ 2_1\ 2_1$	$P\ 2_1\ 2_1\ 2_1$
Unit cell<br>a, b, c (Å)<br>α ,β ,γ (°)	 69.4, 82.0, 157.1<br>90, 90, 90	 69.4, 82.3, 157.7<br>90, 90, 90	 68.4, 82.4, 154.6<br>90, 90, 90
Unique reflections	31596 (3040)	50306 (4201)	18743 (1340)
Redundancy	7.0 (6.6)	10.7 (9.8)	10.1 (8.5)
Completeness (%)	99.8 (99.6)	100.0 (100.0)	97.9 (90.1)
Mean I/sigma(I)	12.12 (2.16)	23.99 (4.90)	9.06 (4.11)
R-merge	0.10 (0.70)	0.08 (0.49)	0.23 (0.81)
**Refinement**			
R-work	0.186 (0.252)	0.185 (0.223)	0.194 (0.241)
R-free	0.235 (0.322)	0.228 (0.287)	0.247 (0.321)
Wilson B-factor	42.9	31.5	52.7
Number of non-hydrogen atoms	6505	6749	6330
Macromolecules	6355	6334	6330
Water	150	415	16
RMS(bonds)	0.010	0.009	0.012
RMS(angles)	1.46	1.32	1.45
Ramachandran favored (%)	98	99	99
Ramachandran allowed (%)	2	1	0.74
Ramachandran outliers (%)	0	0	0.26
Clashscore	5.42	5.28	10.07
Average B-factor	45.2	35.6	36.9
Macromolecules	45.2	35.3	36.9
Solvent	43.7	39.9	30

[a]Statistics Rmerge = ∑hkl ∑i| Ihkl, i-<Ihkl> I | / ∑hkl∑i | Ihkl, i |, where <Ihkl> is the mean intensity of the multiple Ihkl, i observations for symmetry related reflections.

[b]Numbers in parentheses are for the highest resolution bin.
